# Supplementary material for: A Likelihood Approach for Real-Time Calibration of Stochastic Compartmental Epidemic Models
Source: PLoS Comput Biol. 2017 Jan 17;13(1):e1005257. doi: 10.1371/journal.pcbi.1005257 (PMC5240920; doi:10.1371/journal.pcbi.1005257)
Supplement: S1 File — (TAR.GZ) [file pcbi.1005257.s014.tar.gz › HSPH_Online-SI-Revision/PF08/n100-mild/n100-mild_PF_summary.pdf]

peak −8	R0	$1/\Theta_2$	inf	inflog	newly	newlylog	Reff	AttackRate
mean	47.	422.5	715.5	0.7	71.	0.2	45.1	81.2
median	46.6	387.9	411.2	0.7	34.6	0.2	44.3	78.8
Quantile .1	35.5	278.7	213.5	0.5	15.8	0.1	35.2	55.5
Quantile .9	55.9	563.7	1747.	1.1	168.6	0.5	53.7	111.5
11	0	11						
length	50							

peak −4	R0	$1/\Theta_2$	inf	inflog	newly	newlylog	Reff	AttackRate
mean	80.5	473.3	421.5	0.7	15.8	0.1	73.1	114.
median	77.6	428.4	388.2	0.7	12.1	0.1	66.8	108.2
Quantile .1	47.3	247.5	248.5	0.5	5.5	0.	42.5	87.9
Quantile .9	117.4	665.2	605.1	0.8	29.1	0.2	108.1	146.1
0	0	0						
length	50							

peak	R0	$1/\Theta_2$	inf	inflog	newly	newlylog	Reff	AttackRate
mean	85.5	500.2	627.3	0.8	43.	0.2	44.8	124.3
median	73.3	433.5	586.9	0.8	41.1	0.1	40.5	117.4
Quantile .1	40.4	250.4	315.7	0.6	10.5	0.	26.	91.8
Quantile .9	132.7	783.	984.3	1.	65.5	0.2	70.4	163.7
0	0	0						
length	50							

peak +4	R0	$1/\Theta_2$	inf	inflog	newly	newlylog	Reff	AttackRate
mean	77.1	483.9	6985.7	1.4	868.7	0.7	17.4	127.5
median	57.4	400.6	1765.1	1.3	356.9	0.7	13.	122.1
Quantile .1	40.7	262.7	868.9	1.	158.2	0.4	2.4	95.1
Quantile .9	129.4	783.	6620.9	1.8	855.7	1.	38.6	170.1
0	0	0						
length	50							

peak +8	R0	$1/\Theta_2$	inf	inflog	newly	newlylog	Reff	AttackRate
mean	77.4	481.9	282 964.	2.	82 228.8	1.4	39.5	128.5
median	56.4	395.4	6756.	1.8	1503.7	1.2	35.5	123.2
Quantile .1	41.4	239.8	2138.8	1.4	450.	0.7	14.9	96.
Quantile .9	132.5	783.	77 055.6	2.9	9997.	2.	65.	167.4
0	0	0						
length	50							
